# Community Pharmacists’ Involvement in Research in the United Kingdom

**DOI:** 10.3390/pharmacy5030048

**Published:** 2017-08-28

**Authors:** Philip Crilly, Nilesh Patel, Abisola Ogunrinde, Doreen Berko, Reem Kayyali

**Affiliations:** 1 School of Life Sciences, Pharmacy and Chemistry, Kingston University, Kingston-upon-Thames KT1 2EE, UK; p.crilly@kingston.ac.uk (P.C.); alogunrinde@googlemail.com (A.O.); doreenberko@hotmail.com (D.B.); 2 School of Pharmacy, Reading University, Reading RG6 6AP, UK; Nilesh.Patel@reading.ac.uk

**Keywords:** community pharmacy, pharmacy research, pharmacy services, evidence-base

## Abstract

**Puropse**. To investigate the engagement of community pharmacists (CPs) with pharmacy research and identify barriers preventing them from doing so. In addition, to determine the training and research tools available to support CPs to take part in research. **Methods**. A questionnaire was designed and distributed to a sample of community pharmacies (*n* = 323) within five local authorities in England, and to a random sample of community pharmacies (*n* = 329) within Greater London in two stages. Descriptive statistics were used to analyse the data using Microsoft Excel. Following questionnaire completion, CPs were invited to take part in face-to-face and telephone interviews to further explore their views on research. Interviews were transcribed and analysed using coding and thematic analysis. **Results**. A total of 104 questionnaires were completed out of 652 distributed. Over half (56.7%) of respondents considered research to be important to their practice. Approximately 88% of respondents had completed some form of mandatory research in the past two years, while only 29% were involved in non-mandatory research. Over two-thirds (67.9%) wanted to engage with research in the future, with 22.2% of these being most interested in recruiting patients for research. Barriers to research included lack of time (90%) and lack of remuneration (60%). 20 community pharmacists were interviewed. Three themes were identified: 1. Interest in taking part in research; 2. Awareness, support and knowledge; 3. Resources as barriers. **Conclusion**. CPs recognise the importance of research in their current practice, however, the biggest barrier they face is time. Further training may be useful to ensure CPs are adequately prepared to undertake research activities.

## 1. Introduction

In the UK, community pharmacists (CPs) are relied upon to provide evidence-based and cost-effective support to the public and other healthcare professionals [1].

Pharmacists deliver their role as part of a National Health Service (NHS) contract. As the NHS becomes more cost conscious, it needs reassurance that pharmacy initiatives are value for money and backed by robust research [2,3].

The Pharmacy in England report states, “… for [a] pharmacy to be recognised as a key and essential element in the delivery of clinical services, a sound evidence base that demonstrates how pharmacy delivers effective, high quality and value for money services is needed” [4]. The report acknowledged the value of practice research and found a number of studies, mainly overseas, that strongly support the services run in community pharmacies [5]. It concluded that there is a demand for research to be done in the UK that can directly correlate with pharmacy practice.

Clinical Commissioning Groups (CCGs) and Local Authorities (LAs) are NHS bodies that are responsible for commissioning health services in the areas in which they serve [6]. They base their decisions on three factors: resources, value, and evidence [7] to show efficiency, quality, and performance. Pharmacy practice research (PPR) is thus essential in showcasing an evidence base to protect, justify, and develop pharmacy roles and services [8]. It tries to “understand pharmacy ... to ensure that pharmacists’ knowledge and skills are used to best effect in solving the problems of the health service” [9,10]. PPR has been a facilitator for services implemented through pharmacy, for example, smoking cessation service, [5] repeat dispensing service [11], and advanced pharmacy services: medicine use reviews (MURs—supporting patients on multiple medicines to improve adherence) and new medicine service (NMS—supporting patients on newly prescribed medicines to improve adherence) [12,13].

For pharmacists interested in research, the Royal Pharmaceutical Society (RPS) introduced a Research Resource Hub in July 2016. The aims of the hub are to inspire pharmacists to become involved in research and to provide support to those keen on developing this aspect of their career [14]. Charities such as Pharmacy Research UK [15] aim to build pharmacy research capacity and capability through grants, training bursaries, and post-graduate research funding.

Apart from a short research report, [16] a recent systematic review [17] highlighted that the last published study of UK-based community pharmacists’ perceptions of research was in 2000 [18]. Two of the studies that explored pharmacists’ attitudes towards research, in the UK in 2000 [18] and Australia in 2009 [19], suggested that pharmacists do realise that research is valuable to the profession. Interestingly, 33% of pharmacists in the Peterson et al. [19] study and 43.2% of pharmacists in the Rosenbloom et al. [18] study had previously done some research. The main barrier to research in the latter study was the fact that no payment was received for the work done, while in the former study, the main barriers were lack of time and lack of notification about research priorities.

Recent threats of cuts to pharmacy funding have hastened the need to encourage more CPs to become involved in research, to showcase how they can deliver positive and lasting health outcomes for a new NHS. This study aimed to determine the level of CP engagement with research in England, to explore the resources and topics that can act as facilitators for their engagement, and the barriers that prevent them from participating in research.

## 2. Materials and Methods

The study employed a mixed method approach in the form of a survey followed by interviews. A questionnaire was designed consisting of 20 questions in five sections: Community pharmacists and the importance of research; Past and current engagement in research; Future engagement in research; Facilitators and barriers to research; and Demographics (no identifiable data required). A brief definition of research and its different types (Table 1) was provided as part of the questionnaire. These definitions were obtained from the Royal Pharmaceutical Society (RPS) research and evaluation hub [14].

There was also an invitation to take part in an interview to further explore attitudes on practice research. An interview schedule was devised to conduct short semi-structured interviews (Table 2).

The delegated ethical approval team operating under the Kingston University Science, Engineering and Computing faculty ethics committee granted ethical approval for the survey on 14 January 2013 and the interview schedule on 27 March 2013 (1213/045).

The study was carried out in two stages over two years (2013 and 2014). The first stage addressed pharmacist perceptions across England. Stratified sampling was used to divide England into 5 major regions: North of England, East of England, London (South of England), Welsh Borders and West Country (South West England), and the Midlands. Each region was then divided into 4 areas: north, south, east, and west. Random sampling was used to select a county and a local authority within each of the 5 areas selected. The local authorities selected for this study were: Bedfordshire, Cornwall and Isles of Scilly, Richmond and Twickenham, and Wakefield and Warwickshire. The total number of pharmacies was identified in each local authority. A sample size for each local authority was calculated using a Raosoft sample size calculator [20], providing a confidence level of 95% and a margin of error of 5% (Table 3). The total sample size calculated for all five local authorities was 323. 

The second stage of the study addressed perceptions of pharmacists working in community pharmacies in Greater London (*n* = 2250) [21]. A sample size of 329 was calculated using a Raosoft sample size calculator [20], providing a confidence level of 95% with a margin of error of 5%. In both stages of the study, an assumption was made that there would be one pharmacist working in each pharmacy. Pharmacies within the research area were assigned a number; this was then randomised using an online randomisation tool.

Questionnaires were distributed by post, with stamped addressed envelopes included to encourage their return. To enhance the response rate, pharmacists in the local area (Richmond and Twickenham) were also approached in person to complete the survey. In addition, an online version of the questionnaire was created in Google Drive and sent via email to pharmacies who had not responded within 2 weeks. Their email addresses were obtained from the NHS Choices website [21]. All CPs were given a participant information sheet and were given two weeks to return their questionnaire. Pharmacists who had not responded by the deadline were telephoned to check they had received the information and asked if they were still interested in participating in the study. Completion of the questionnaire was accepted as informed consent.

All responses were entered into a Microsoft Excel spreadsheet and analysed using descriptive statistics.

Following completion of the questionnaire, CPs who indicated that they were willing to participate in the second phase of the study were invited for interview. Having established pharmacists’ interpretations of research and their understanding of the different types of research through the questionnaire, the aim of the interview was to explore pharmacists’ involvement in research and the barriers they faced. The interview schedule was piloted by 5 community pharmacists. No changes were recommended. Interviews were to be conducted until a saturation of themes had been achieved. Greater London was chosen due to its convenience of access to conduct interviews.

The interviews were conducted by the female authors AO and DB, as part of their integrated Masters qualification. Both researchers received interview skills training at the university. In advance of the interview, the interviewee was given a participant information letter and consent form to sign. Interviews were carried out in either the private consultation room at the place of work of the CP, with only the interviewer and interviewee present, or over the telephone. These took place at a time, indicated by the CP, which would be least likely to have interruptions. Each interview took approximately 20 min to complete. These were digitally audio-recorded with the permission of the interviewee. Written transcripts of each recording were prepared and participants were asked to comment on their accuracy. The transcripts were analysed using coding and thematic analysis in NVivo qualitative data analysis Software, version 11 (QSR International Pty Ltd., Melbourne, Australia). This was done using inductive (from data) and deductive (from literature) approaches. The transcripts were read and re-read several times, and coded manually and independently by two researchers. Thereafter, the codes were checked by a third researcher and discussed by all researchers to ensure consistency of findings. Results are presented as themes with quotes from interviews used to support these.

## 3. Results

A total of 104 questionnaires were collected out of the 652 posted and hand delivered over both stages of the research, giving a response rate of 16.0%. The breakdown of how many responses were received from each local authority is shown in Table 3. The sample demographics (*n* = 104) are shown in Table 4. In addition, Table 5 highlights community pharmacist perceptions towards practice research.

### 3.1. Community Pharmacists (CPs) and the Importance of Research

Answered using a 5-point Likert scale, over half (56.7%) of the respondents considered research to be important to their current practice and 45.3% agreed that research should be a priority. Over a quarter (26.4%) of pharmacists didn’t perceive research to be part of their role and just over one tenth (13.5%) considered research to be unimportant. 

Almost two-thirds (60%) felt that they had the necessary skills to do research, with 38% stating that they would be prepared to make time for research during their working hours. The majority (86.8%) agreed that research is vital to the development of new pharmacy services, but only under a third (30.2%) felt that there were plenty of opportunities for community pharmacists to take part in research (Table 5). 

### 3.2. Past and Current Engagement in Research

Approximately 88% of CPs surveyed had completed an audit within the past two years and nearly two-thirds (62.7%) had taken part in a service evaluation in the same period.

In relation to non-mandatory research, i.e., research that is not part of the NHS pharmacy contract, 33.3% of CPs had not participated in any within the past two years. Meanwhile, 29% were involved in research which included clinical trials, applied health research, and clinical research. 

### 3.3. Future Engagement in Research

Over two-thirds (67.9%) of CPs specified that they would like to engage in research in the future. There were differences depending on types of employer. Whereas 61% of CPs working in a multiple chain pharmacy wanted to engage in research, only 38.9% of those in independent pharmacies were interested. 

Over three quarters (77.8%) in the 20–29 age categories wanted to engage in research, compared to 35.3% of 50–59 year-olds who wanted to engage in research. 

For CPs interested in research, 22.2% were interested in recruiting patients for research with the same percentage interested in completing audits and service evaluations. Research topics that CPs wanted to engage with included diabetes (41.2%), medicines management (29.4%), and cardiovascular disease (17.6%). 

### 3.4. Facilitators and Barriers to Research Participation by CPs

Barriers to completing research included lack of time (90%), lack of remuneration (60%), and insufficient training (45%). 

Over two-thirds (69%) thought training tools would help facilitate research, whilst 52% thought protected time for research activities would be beneficial. 54.9% of CPs wanted to learn more about data collection and research tool design. 

### 3.5. Interviews

Of the 104 pharmacists who completed the survey, 12 were interviewed over the telephone and 8 were interviewed at their place of work. The sample demographics (*n* = 20) are shown in Table 6.

Interviews were carried out until data saturation was reached [22]. The stopping criterion for data saturation, which is the number of interviews conducted without any new information after which recruitment was stopped, was six. 

Three key themes emerged from the analysis:

- Interest in taking part in research
- Awareness, support, and knowledge
- Resources as barriers

#### 3.5.1. Interest in Taking Part in Research

Pharmacists reported being interested in research activities. They would like to be involved in research that was related to their day-to-day practice, particularly in relation to health promotion and patient care. This was typified in the following examples:

“Looking at community pharmacy research … it would have to be based around factors that improve patient care … health promotion … public health issues.”

(#1)

“I am interested in research … it’s only through research that you are more current and can give better advice …”

(#6)

#### 3.5.2. Awareness, Support, and Knowledge

The pharmacists felt they lacked the necessary knowledge to take part in research. They were also unaware of the areas of interest for community pharmacy research. They felt that organisations such as the Primary Care Research Network (PCRN) and Local Pharmaceutical Committees (LPCs) could do more to promote engagement in research and to encourage pharmacy contractors to work together on research priorities:

“… I don’t see a lot of that coming from our LPC. And they’ve never asked to see what audits and things that we’re doing and saying, ‘Let’s collaborate and get all that information together’ which would be a nice situation but there’s a lot of politics involved in that as well, so …”

(#10)

Pharmacists reported that if management were supportive of research, then they would be more likely to become involved:

“Big companies … need to put research on their agenda … in that way it will filter down to the pharmacist.”

(#12)

Some felt that had they learned more about research skills at university they would be more receptive to taking part in it:

“… if these things were started off earlier on in our career, you would tend to carry on with it. I think within university if they changed the degree slightly, maybe have more input with drug companies and research then maybe we could be more inclined to get involved.”

(#3)

#### 3.5.3. Resources as Barriers

Finding time for research was of concern to many. Some felt that adding research to their workload would over-burden them when they are already busy:

“I mean … doing evidence-based research is something that everybody would like to do… the reality is … do you have enough time in your day?”

(#7)

The prospect of remuneration for the time needed to take part in research was appealing to some pharmacists:

“If it is incentivized … I will be more likely to be involved in research.”

(#4)

Suggestions were made that pharmacy support staff could help the pharmacist with research activities:

“My staff, if I tell them what to do … yes they have the capabilities to help.”

(#19)

In addition, lack of appropriate infrastructure was identified as a barrier to research:

“One of the hardest things is when we receive information it’s quite difficult to fit it in with the IT because it quite often comes in standalone project. If it had been computer based it would have been a lot easier and obviously time is a big factor”

(#16)

## 4. Discussion

This study has highlighted that community pharmacists are interested in taking part in research activities, believing that research improves patient care. In fact, the findings from the questionnaires showed that more than half of the CPs surveyed considered research to be important to their current practice. These views were similar to those held by CPs in other studies [17,18,19,23]. Some CPs even went so far as to express concern that pharmacy services would be cut if it could not be proved by research to be effective. This is because the model of service commissioning is dependent on performance [1]. The performance of these services can only be determined by pharmacy research, so if CPs do not participate, then there will be no evidence to support the efficiency of services provided.

Nevertheless, most research in community pharmacy appears to be the completion of mandatory tasks such as audits and service evaluations with 88.2% of respondents having completed one or the other within the last two years. The numbers dropped sharply when it came to non-mandatory research. These findings indicate that CPs are prioritising mandatory research as it is part of their contractual framework with some suggesting that involvement in non-mandatory research is for academics. This is supported by another study, [9] which states that CPs still consider research to be reserved for academic institutions. Other reasons given for lack of involvement included lack of time, no remuneration, the workload, lack of support from management, and the fact that they were not approached. Similar findings were noted by Elkaseem et al. [23] when evaluating pharmacist experiences in Qatar. Additionally, some CPs lacked confidence and the resources required to carry out research. These observations were similar to those held by Daly [24], who described a project where eight pharmacists were appointed as part time community pharmacy research facilitators to establish a network of interested pharmacists who would contribute to research. She reported that “many pharmacy teams assumed that … research was time consuming and onerous” [24]. CPs felt that remuneration was the biggest incentive to facilitate research followed by training tools, protected time, and support from management. 

Lack of appropriate infrastructure to do research was listed as another barrier with CPs, suggesting that management should put research on their agenda. They felt that appropriate infrastructure would include guidance, training, and notification of research topics. The RPS hopes that with its Research and Evaluation Hub, this will become a valuable resource for pharmacists hoping to learn the skills required to become involved in research [14]. However, more awareness of such support is needed. In regards to the role CPs wanted to play in research, the most commonly suggested role was recruiting patients for research, as well as taking part in audits and service evaluations.

Of note, younger pharmacists appeared to be more interested in taking part in research than their older counterparts. This could be due, in part, to their more recent experience of completing an academic research project in their MPharm course. A study by Langley et al. [25] noted that almost two-thirds (61%) of final year MPharm students considered their research project to be very important or fairly important, a perception that may continue into their early career. Three pharmacists in the interviews, however, felt that undergraduate pharmacy programmes needed to be even more focused on research, hypothesising that if this was the case then future pharmacists’ skills could be built upon from an early stage in their career and engagement in research would become the norm. Professor Dean Franklin of the Modernising Pharmacy Careers Programme board states, “Research is like an apprenticeship; you can only learn by doing it but, unless you have the opportunity to start doing it, it’s very difficult to develop the necessary skills and expertise” [26]. 

The findings of this study add to the outcomes of similar studies [27,28] in countries other than the UK and demonstrate an emerging trend for greater community pharmacist involvement in research [23].

Limitations of this study include a low response rate, meaning broad generalisations could not be made. Although random selection was used, selection bias may have occurred with the participation of those more interested in research. The authors’ assumption that one pharmacist worked in each pharmacy may be inaccurate for some pharmacies; this may mean that our sample is not representative of all pharmacist opinions. In addition, as this research was carried out in 2014, there is a possibility that community pharmacists may feel differently about research following the launch of the new Research and Evaluation Hub by the RPS. 

## 5. Conclusions

Active involvement in pharmacy research has been acknowledged by community pharmacists in this study as being important to the future of the profession. Practice research can help community pharmacy to conform within the NHS reforms.

Many pharmacists would like to become more involved in research, but feel that factors like lack of training and lack of employer support limit any research they could do. More training in research techniques, as well as more promotion of pharmacy research priorities, would help to ensure that more pharmacists contribute to essential research projects and showcase the profession as being at the forefront of evidence-based practice. 

## Figures and Tables

**Table 1:** Types of research conducted in community pharmacy.

**Mandatory Research**	
Community Pharmacy Patient Questionnaire	Obtaining patient perceptions of the service provided by a community pharmacy
Clinical audit	A tool to compare how a pharmacy service is delivered based on pre-agreed standards
Non-mandatory research	
Service evaluation	Reviewing whether a service is meeting the needs of the patients
Clinical trials	A way to find out how effective an intervention is
Applied health research	Research that can be applied to a real problem
**Research Methods and When They Are Used [14]**	
Survey (Quantitative)	Efficient way of obtaining information from a large number of respondents
Interview (Qualitative)	To gain an understanding of underlying reasons and motivations—participants can give in-depth reasons and explanations
Focus group (Qualitative)	Similar to interview but in addition, allows discussion and forming of opinions in a natural interactive process

**Table 2:** Interview schedule used to interview community pharmacists (CPs) about their opinions of CP involvement in pharmacy research.

Interview Schedule
Are you aware of the current push for research and the research ready tools?
Are you a member of the RPS?
Which sources, sites and avenues could be used to push research and be accessed by pharmacists?
What are some of the barriers to research?
Have you participated in any research yourself?
What research projects would you be interested in doing in the future?
What sort of training have you received to help you in taking part in research?
What sort of training tools do you think would help pharmacists engage in research?
What are some of the facilitators for research?
What level would you like to engage in research?

**Table 3:** Sample calculated and determined using Raosoft sample size calculator.

	Local Authorities				
	Posted				Hand Delivered
	Bedfordshire	Cornwall and Isles of Scilly	Wakefield	Warwichshire	Richmond and Twickenham
**Total number of pharmacies**	70	96	73	104	45
**Recommended sample size**	60	77	62	83	41
**Responses recieved**	15	26	20	19	24

**Table 4:** Sample characteristics (*n* = 104).

Characteristic	N (%)
Gender	Female 55 (53%)
	Male 49 (47%)
Type of employer	
Multiple pharmacy (>10)	67 (64%)
Small multiple pharmacy (2–10)	8 (8%)
Independent pharmacy	29 (28%)
	Average Years
Experience as a pharmacist	13

**Table 5:** General perceptions of community pharmacists (*n* = 104) towards practice research.

Statements	Strongly Agree	Agree	Neither Agree nor Disagree	Disagree	Strongly Disagree
	%	%	%	%	%
There is an abundance of opportunities for community pharmacists to take part in research	3.8	26.4	24.5	34	11.3
Community pharmacists have the necessary skills to do research	17	43	20.8	17	1.9
I would be prepared to make time for research during my working hours	5.7	32.1	20.8	26.4	15.1
Research is fundamental to the future of the pharmacy profession	26.4	49.1	17	7.5	0
My daily activities prevent me from engaging in research	24.5	39.6	20.8	13.2	1.9
I don’t perceive research as part of my role as a community pharmacist	7.5	18.9	28.3	35.8	9.4
Remuneration is an incentive for me to participate in research	37.7	34	17	7.5	3.8
I have the storage and electronic capacity to hold research data in the pharmacy	15.1	22.6	22.6	22.6	17
Research is vital to the development of new pharmacy services	24.5	62.3	7.5	5.7	0
I would engage in research if I had fully capable pharmacy staff for support in the workplace	26.4	54.7	11.3	5.7	1.9
The way I practice has been influenced by research	17	24.5	37.7	17	3.8
Research should be a high priority for community pharmacists	13.2	32.1	37.7	13.2	3.8
It is important that community pharmacists are kept informed of research relevant to the practice of pharmacy	30.2	54.7	13.2	1.9	0
If I knew my competitors were involved in research, I would want to get involved too	7.5	37.7	34	13.2	7.5

**Table 6:** Demographics of pharmacists interviewed (*n* = 20) about their views on research.

		Telephone Interviews (*n* = 12)	Face-To-Face Interviews (*n* = 8)
**Gender**	Male	6	3
	Female	6	5
**Age**	20–29	4	2
	30–39	5	3
	40–49	2	2
	50–59	1	1
**Type of community pharmacy**	Independent	3	5
	Small multiple	1	2
	Large multiple	8	1
